# The Microbial Network Stability in Cyanobacterial and Moss Biocrusts Respond Differently to Climate Warming 

**DOI:** 10.3390/microorganisms14030713

**Published:** 2026-03-22

**Authors:** Chang Tian, Chongfeng Bu, Shufang Wu, Xinhao Li, Kadambot H. M. Siddique

**Affiliations:** 1 Bio-Agriculture Institute of Shaanxi, Xi’an 710043, China; 18531395352@163.com; 2 Enzyme Engineering Research Center of Shaanxi, Xi’an 710600, China; 3 State Key Laboratory of Soil and Water Conservation and Desertification Control, Northwest A&F University, Yangling 712100, China; lixinhao0327@163.com; 4 Institute of Soil and Water Conservation, Chinese Academy of Sciences and Ministry of Water Resources, Yangling 712100, China; 5 Institute of Water Saving Agriculture in Arid Areas of China, Northwest A&F University, Yangling 712100, China; wsfjs@163.com; 6 College of Water Resources and Architectural Engineering, Northwest A&F University, Yangling 712100, China; 7 The UWA Institute of Agriculture, The University of Western Australia, Perth, WA 6001, Australia; kadambot.siddique@uwa.edu.au

**Keywords:** biocrusts, climate warming, microbial network stability, microbial network complexity, semi-arid regions

## Abstract

Climate warming—a key driver of global change—significantly affects soil microbial communities and network stability. Biological soil crusts (biocrusts) help mitigate these impacts while maintaining soil ecological functions and biodiversity. However, how microbial networks and community dynamics respond to warming remains poorly understood between biocrust types, namely cyanobacterial and moss biocrust. In this study, we investigated the effect of warming on microbial communities and network stability in these biocrusts within the Mu Us Sandland, China. Using structural equation modeling (SEM), we found that warming altered microbial network properties: compared to the control, warming increased network vulnerability and decreased robustness specifically in cyanobacterial biocrusts. Warming and decreased soil moisture acted as strong filtering factors, resulting in lower microbial network stability. Although overall network complexity remained unchanged, warming reduced connectivity in cyanobacterial biocrusts, thus undermining network stability. Moreover, under both warming and control conditions, moss biocrusts exhibited lower robustness but higher vulnerability than cyanobacterial biocrusts, indicating cyanobacterial biocrusts displayed greater microbial network stability in comparison. Additionally, warming reduced the number of module hubs and keystone phyla in both biocrust types, decreasing key taxa abundance and weakening direct microbial interactions. We concluded that warming impaired microbial network stability by reducing connectivity in cyanobacterial biocrusts. These findings highlight the superior capacity of cyanobacterial biocrusts to sustain soil microbial network stability under climate warming and identify shifts in network connectivity as a central mechanism driving biocrust responses to environmental stress.

## 1. Introduction

Global temperatures have risen by approximately 0.8 °C over the past century, with projections indicating an increase of 1.5–4 °C by the end of this century [1]. Climate warming is a key driver of changes in terrestrial ecosystems—including shifts in plant distributions [2] and in soil microbial community composition and diversity [3]—with broad implications for biodiversity, ecological balance, and function [4,5,6,7]. Soil microbes play essential roles in the carbon cycle, regulating processes of carbon mineralization and stabilization [8,9]. Consequently, shifts in microbial communities can significantly influence soil greenhouse gas emissions [10]. Understanding how global change factors affect soil microorganisms and the mechanisms underlying these effects is, therefore, a central question in microbiology and global change research [11], given their fundamental importance for ecosystem functioning [12].

Co-occurrence network analysis is a powerful tool for studying microbial interactions, allowing assessment of community complexity and stability [3,13]. Soil microbes rarely exist as isolated populations; instead, they form intricate and dynamic interaction networks [13]. Climate warming disrupts these interactions, often reducing network connectivity and stability, thereby affecting broader ecosystem functions [14]. For example, warming can indirectly affect microbial networks by altering soil moisture, temperature, and nutrient availability [15]. Studies have shown that warming may reduce microbial network connectivity, decrease keystone species abundance, and weaken overall network stability [14]. However, most existing research focuses on forest and grassland systems, with comparatively little known about the effects of warming on microbial networks in arid and semi-arid ecosystems.

Biological soil crusts (biocrusts)—comprising bacteria, archaea, fungi, algae, lichens, and their secretions—dominate soil surfaces in arid and semi-arid regions [16,17]. These communities contribute to essential ecological processes, including nutrient cycling, soil formation, and water retention [18,19,20,21,22]. Microbial secretions, particularly extracellular polysaccharides, bind soil particles into stable aggregates [23]. The complex symbiotic and competitive relationships among these microorganisms underpin ecosystem energy flow and nutrient cycling [3,24]. Network analysis of microbial communities allows for the identification of keystone taxa that influence community composition and function [25,26]. Understanding microbial interactions and diversity provides critical insights into the structure and function of biocrusts, enhancing our ability to predict their responses to environmental change [24]. In this context, interaction networks complement traditional diversity metrics by offering a more nuanced understanding of community organisation and resilience.

Ecological networks describe species interactions and ecosystem dynamics [3]. A fundamental, yet debated, question concerns whether and how ecological network complexity influences ecosystem function [27]. Compared to bare soils, biocrusts offer more favorable environments for microbes and can buffer the effects of climate change [28]. Warming alters microbial composition and diversity during biocrust development, with potential consequences for ecosystem function [29,30]. Greater microbial diversity is generally associated with enhanced ecosystem stability due to functional redundancy and complementarity [31]. Recent studies document changes in ecological networks and ecosystem functions along environmental gradients or following disturbance [3,32,33]. While an altered microbial network structure affects ecosystem function in warmed forests and grasslands [3]. However, the mechanisms by which warming affects microbial interactions in biocrusts remain underexplored.

This study aims to address this knowledge gap by investigating how warming affects microbial networks in different biocrust types. Specifically, we (1) compare the effects of warming on physicochemical properties across cyanobacterial and moss biocrusts, (2) examine changes in microbial community and network properties, and (3) assess simulated warming responses. Employing high-throughput sequencing and ecological network analysis, we constructed molecular ecological networks for biocrusts in the Mu Us Sandland under simulated warming conditions. We tested two hypotheses: (1) warming reduces microbial network complexity, connectivity, and stability in cyanobacterial and moss biocrusts; (2) warming reduces the number of module hubs and keystone phyla in both biocrust types.

## 2. Materials and Methods

### 2.1. Study Site

**Figure 1:**
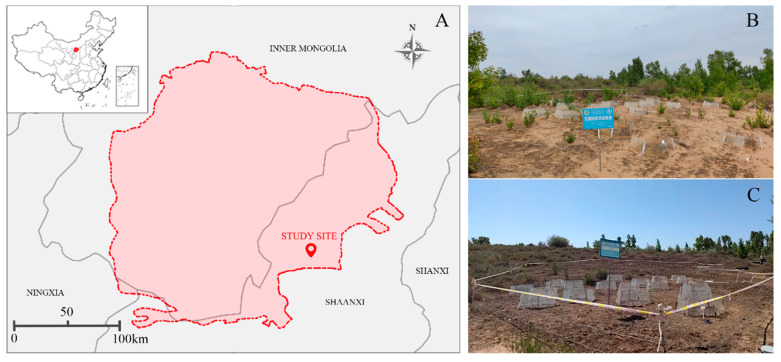
(**A**) Location of the study site and photographs of the sample plots: (**B**) cyanobacterial biocrust experimental plot; (**C**) moss biocrust experimental plot.

The study was conducted at the southeastern edge of the Mu Us Sandland, Hengshan District, Yulin City, Shaanxi Province, China (108°56′41”–110°01′48” E, 37°21′43”–38°14′53” N; see Figure 1A). The region experiences a temperate semi-arid continental monsoon climate, with a mean annual temperature of 8.1 °C and average annual precipitation of 287 mm, approximately 62% of which occurs between July and September [34]. The sandy soils are highly susceptible to wind erosion and have an average bulk density of 1.5 g cm^−3^ [35]. Vegetation comprises sparse shrubs including *Artemisia ordosica*, *Hedysarum mongolicum*, *Hedysarum scoparium*, *Salix psammophila*, and *Agropyron cristatum*. Biocrusts—particularly cyanobacterial and moss types—occupy inter-shrub spaces and cover more than 20% of the soil surface [36,37,38]. Sampling plots were established in May 2021 in areas with well-developed and minimally disturbed cyanobacterial and moss biocrusts. In these plots, the morphological characteristics of cyanobacterial and moss biocrusts, including coverage, thickness, appearance, and dominant species, have been described in our previous studies [39].

### 2.2. Experimental Design and Biocrust Sampling

A factorial design was used to simulate future climate scenarios, combining two biocrust types (cyanobacterial and moss biocrust) and two temperature regimes (non-warming and warming) (Figure 1B,C). The diagram of the experimental process is presented in Figure S1. Each treatment combination had fifteen replicates, and the experiment commenced in May 2021.

Warming was simulated using thirty hexagonal open-top chambers (OTCs) constructed from acrylic panels (60 cm side length, 0.94 m^2^ surface area), set at a 60° incline. Two groups of warming OTCs, consisting of fifteen OTCs each, were divided into cyanobacterial and moss biocrust, respectively. These chambers increased surface temperatures by an average of 1.13 °C, consistent with projected future warming scenarios [40,41,42]. Meanwhile, thirty control plots (1 m × 1 m square) were maintained under ambient conditions. Two groups of non-warming plots consisting of fifteen plots each were divided into cyanobacterial and moss biocrust, respectively.

In October 2022, surface litter was removed from each plot, and three biocrust samples with well-developed and minimally disturbed biocrust were randomly collected from each plot using a sterile cutting ring (5 cm diameter) and composited into one sample. Samples were divided for separate analyses: one portion (about 250 g) was stored at 4 °C for soil functional measurements, and the other (about 250 g) at –80 °C for DNA extraction.

A polyvinyl chloride (PVC) collar (20 cm inner diameter, inserted 2 cm into the soil) was installed at the center of each sampling plot. This collar was designed to couple directly with an external PVC pipe (20 cm outer diameter, 20 cm height, with a sealed top and open base) used for measurement. Soil respiration was measured monthly from May to October across the 2021–2022 study period. Measurements were taken at each installed collar using a Li-820 automated soil CO_2_ efflux system (Li-Cor, Lincoln, NE, USA). All living plants and litter within the collars were removed 24 h prior to each measurement. Sampling was conducted between 09:00 and 12:00 local time.

Soil pH was measured in a 1:2.5 (*w*/*v*) soil–water suspension using a Leici electrode (Shanghai, China). Soil water content was determined by oven-drying at 105 °C to constant weight. Soil organic carbon was measured using potassium dichromate oxidation [43]. Total nitrogen was determined by the Kjeldahl method, and total phosphorus was quantified using molybdenum blue UV-Vis spectrophotometry following acid digestion [44]. Microbial biomass carbon (MBC) and microbial biomass nitrogen (MBN) were determined using the chloroform fumigation-extraction method [45].

### 2.3. Soil Microbial Molecular Analysis

Microbial community composition was assessed using high-throughput sequencing. The summarised flowchart is presented in Figure S2. Total genomic DNA was extracted from 0.5 g of soil using a PowerSoil DNA Isolation Kit (Mo Bio Laboratories, Carlsbad, CA, USA), and purity and concentration were assessed using a NanoDrop One spectrophotometer (Thermo Fisher Scientific, Waltham, MA, USA). The V4 region of the 16S rRNA gene was amplified using primers 515F/806R (TaKaRa Premix Taq v2.0) under the following thermal cycling conditions: initial denaturation at 95 °C for 5 min; 28 cycles of 95 °C for 45 s, 55 °C for 50 s, and 72 °C for 45 s; followed by a final extension of 72 °C for 10 min. Triplicate PCR products per sample were pooled, visualized on 1% agarose gel, and quantified using GeneTools software (v4.03.05.0). Equimolar amplicons were gel-purified (E.Z.N.A. Gel Extraction Kit) and used to prepare sequencing libraries (NEBNext Ultra II DNA Library Prep). Sequencing was performed by Megigene Company (Shenzhen, China) using the Illumina Nova 6000 platform.

Raw sequences were processed using Trimmomatic (v0.36) and Pear (v0.9.6) for quality filtering and assembly, with criteria of minimum 120 bp read length, quality score > 20 in 50 bp windows, and minimum 10 bp overlap. Chimeric sequences were removed using VSEARCH (v2.7.1). Operational taxonomic units (OTUs) were clustered at 97% sequence similarity using QIIME2 (v2019.4) with VSEARCH (v2.13.4) [46]. To analyse microbial richness and diversity, the number of sequences per sample should be equal. Therefore, we rarefied 38,847 final tags of randomly selected clean tags per sample to correct for differences in sequencing depth. The average percentage of final tags used in downstream analysis was 44.8% (36.5–54.1%) (Table S1) [47]. Taxonomic assignments were made using the SILVA database and the RDP classifier [48,49].

### 2.4. Network Construction and Analysis

Co-occurrence networks were constructed separately for each treatment using fifteen samples per network. Only OTUs detected in at least twelve replicates were retained. Networks were generated using the Molecular Ecological Network Analysis (MENA) pipeline based on Spearman correlation coefficients (|r| ≥ 0.890, *p* < 0.001) following random matrix theory [3,50]. To describe the network, network topology metrics were calculated and visualized using Gephi (v0.10.1). We focused on several indices, including the number of nodes, the number of links, average degree, connectivity (average links per node), average clustering coefficient (the extent to which nodes are clustered), average path length (average distance between any two nodes), density, diameter, betweenness centralization, closeness centrality and modularity (a measure of how well a network is divided into modules). Network modules represent groups of taxa that share similar ecological functions, niches, or phylogenetic traits [51]. Based on within-module (Zi) and among-module (Pi) connectivity thresholds, nodes were classified into four categories: peripherals (specialists with few connections), connectors (generalists linking modules), module hubs (generalists within specific modules), or network hubs (super generalists central to overall network connectivity). Node roles were determined by within-module connectivity (Zi) and among-module connectivity (Pi) thresholds (Zi = 2.5, Pi = 0.62) [52,53].

Network stability was assessed using the vulnerability and robustness scores for each sample [54,55]. Network vulnerability refers to how quickly the biological consequences of ecological events are spread across parts or the entire network. Network robustness is defined as the percentage of remaining species after a certain species becomes extinct. For simulations of random removal, robustness was measured by removing 50% of random nodes from each network. For simulations of targeted removal, robustness was compared when five module hubs were removed and when half of the module hubs were removed, since the number of module hubs differed greatly between networks. The values were calculated according to Yuan et al. [3]. Network complexity was calculated as the average of standardized (min–max normalized) values for betweenness centralization, closeness centrality, average clustering coefficient, density, and diameter [56,57,58,59].

### 2.5. Statistical Analysis

All statistical analyses were performed in R (v4.2.2) using the vegan package. Soil properties and microbial community metrics were compared using one-way analysis of variance (ANOVA), with *p* < 0.05 considered significant. Structural equation modeling (SEM) was conducted in Amos (v23.0) to assess the direct and indirect effects of warming, soil moisture, species richness, Shannon diversity, and network connectivity on network vulnerability. Model fit was evaluated using maximum likelihood estimation [60]. The piecewise structural equation model’s goodness-of-fit was assessed using chi-squared/degrees of freedom ratio (χ^2^ df^−1^ < 3) and AIC, where non-significant values (*p*-value > 0.05) denote a satisfactory model fit [61]. According to Hair et al. [62], a well-fitting model should have CFI and GFI greater than 0.90, while an RMSEA value less than 0.05, supported by a narrow confidence interval.

## 3. Results

### 3.1. Effects of Warming on Physicochemical and Microbial Community Characterisations

Warming primarily impacts soil microclimate by reducing soil moisture. As expected, SWC results significantly decreased in both biocrusts under warming (Table S2). Similarly, TN, MBC, and MBN also decreased significantly in cyanobacterial biocrust. In contrast, the warming treatment increased pH. SOC and TP did not show any significant differences. However, SOC, TN, and TP in the samples increased in a stepwise manner in moss biocrust. There were no significant differences in soil pH, MBC, and MBN between the warming and control treatments.

**Figure 2:**
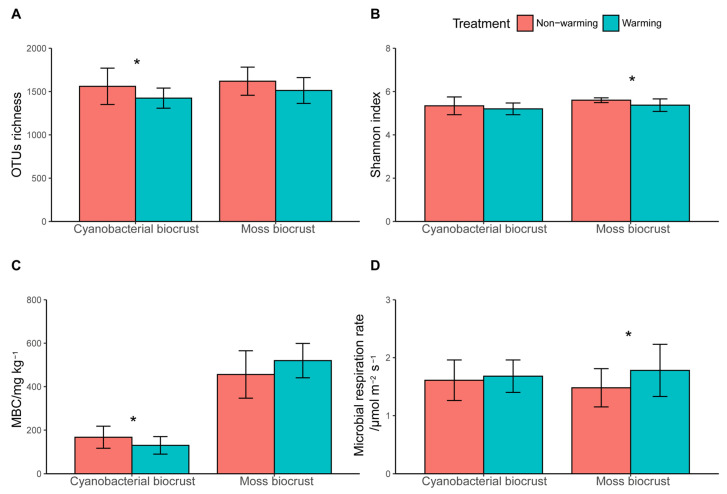
Microbial (**A**) richness and (**B**) diversity, (**C**) microbial biomass carbon, and (**D**) microbial respiration rate response in cyanobacterial and moss biocrusts. Columns represent the mean for each treatment; error bars represent standard deviation (*n* = 15). * indicate significant differences within groups (*p* < 0.05).

The richness of prokaryotic microorganisms in cyanobacterial biocrust was significantly lower under warming treatment, and the same trend was observed for the diversity of prokaryotic microorganisms in moss biocrust (Figure 2). The effect of warming on the abundance of prokaryotic microorganisms in moss biocrust and the diversity of cyanobacterial moss was found to be insignificant. Furthermore, warming significantly increased microbial respiration rate by 0.30 μmol m^−2^·s^−1^.

### 3.2. Responses of Microbial Network Properties

All connectivity curves conformed well to the power-law distribution (R^2^ = 0.91–0.93; Table 1), indicating scale-free network structures where most nodes had few connections and a small number of nodes acted as highly connected hubs [63,64]. The average path lengths (7.99–10.81) were consistent with logarithmic scaling based on network size, indicating that all networks exhibited small-world properties, enabling efficient information flow. High modularity values (0.85–0.96) further indicated that each network could be separated into multiple modules (values > 0.4 were used as the threshold to define modular structures).

Warming had different effects on the network attribute characteristics of the two types of biocrusts. Specifically, warming increased network vulnerability in both biocrust types, with a greater increase in cyanobacterial biocrusts (Table 1). In cyanobacterial biocrusts, warming reduced the number of nodes from 728 to 672 and the number of links from 959 to 763 (Table 1). The average path length increased from 7.99 to 10.81, while network connectivity declined by 44.35%. Both modularity and vulnerability increased by 7.50% and 70%, respectively. In moss biocrusts, warming decreased the number of nodes from 652 to 627 and the number of links from 645 to 584. The average path length increased by 4.88%, while connectivity declined by 25.42%. Modularity and vulnerability increased by 1.27% and 13.33%, respectively. All networks contained more positive than negative links, but the proportion of positive links declined under warming, from 64.96% to 57.83% in cyanobacterial biocrusts and from 64.88% to 58.05% in moss biocrusts. Moreover, for the cyanobacterial biocrust, network robustness showed an opposite trend to vulnerability, with higher values observed in ambient conditions than in the warming treatment. However, moss biocrust networks under warming treatment exhibited higher network robustness (under both random and targeted removal) than under ambient conditions. No significant difference was found among treatments for network stability indexes.

The biocrust type also had different effects on the network attribute characteristics. Moss biocrusts exhibited lower nodes, links, average degree, clustering coefficient, density, connectivity, and robustness, but higher modularity and vulnerability than cyanobacterial biocrusts.

### 3.3. Keystone Microbial Taxa, Module Hubs, and Connectors in Response to Warming

**Figure 3:**
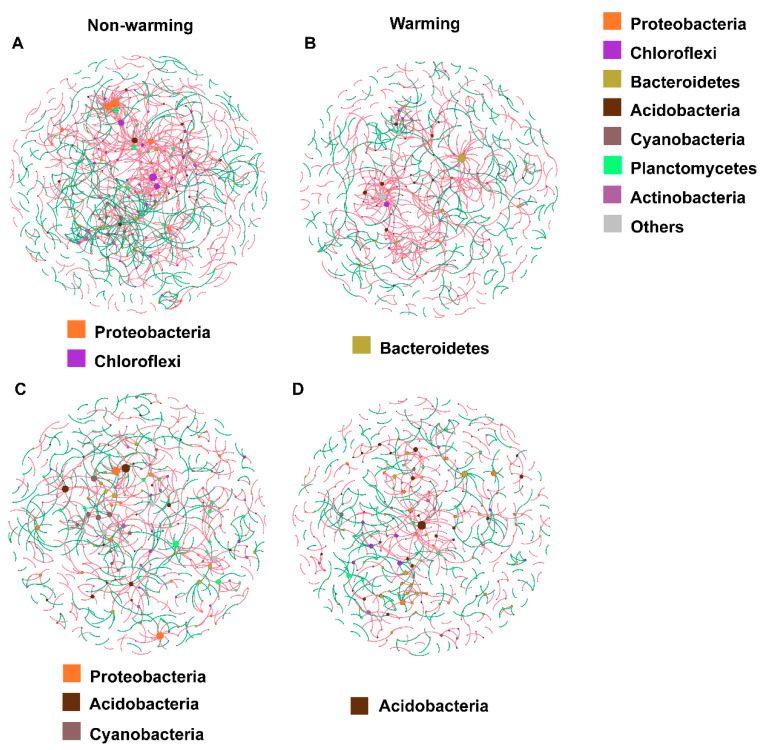
Overview of microbial co-occurrence networks in (**A**,**B**) cyanobacterial and (**C**,**D**) moss biocrusts. Node size corresponds to node degree. Node color represents phylogenetic phyla. Red lines indicate positive interactions, while green lines indicate negative interactions.

Warming altered the composition of keystone phyla in cyanobacterial biocrusts and reduced the overall number of keystone phyla in moss biocrusts. Five bacterial phyla were present across all networks, but their composition varied by treatment (Figure 3). In cyanobacterial biocrusts, Chloroflexi and Proteobacteria were keystone taxa under ambient conditions (Figure 3A), whereas Bacteroidetes became more prominent under warming conditions (Figure 3B). In moss biocrusts, Proteobacteria and Cyanobacteria were dominant under ambient conditions (Figure 3C), but Acidobacteria emerged as a key phylum under warming (Figure 3D).

**Figure 4:**
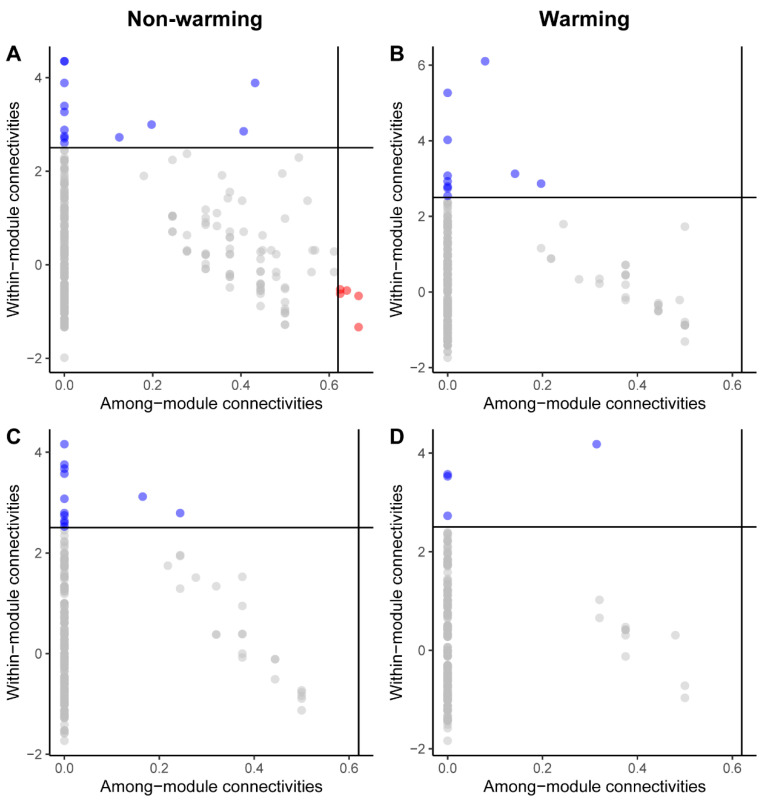
Zi-Pi plots showing the distribution of operational taxonomic units (OTUs) based on their topological roles within microbial networks of (**A**,**B**) cyanobacterial and (**C**,**D**) moss biocrusts. Each dot represents an individual OTU. Peripherals, connectors, and module hubs are represented by gray, red, and blue dots, respectively.

Warming reduced the number of module hubs in both biocrust types, with a more pronounced decline observed in moss biocrusts. No network hubs were identified in any treatment (Figure 4). In cyanobacterial biocrusts, the non-warming network contained 13 module hubs and 5 connectors, with 98.36% of nodes classified as peripherals. Under warming, the number of module hubs decreased to 10. In moss biocrusts, module hubs declined more substantially, from 12 under ambient conditions to 4 under warming.

### 3.4. Structural Equation Models Analysis

**Figure 5:**
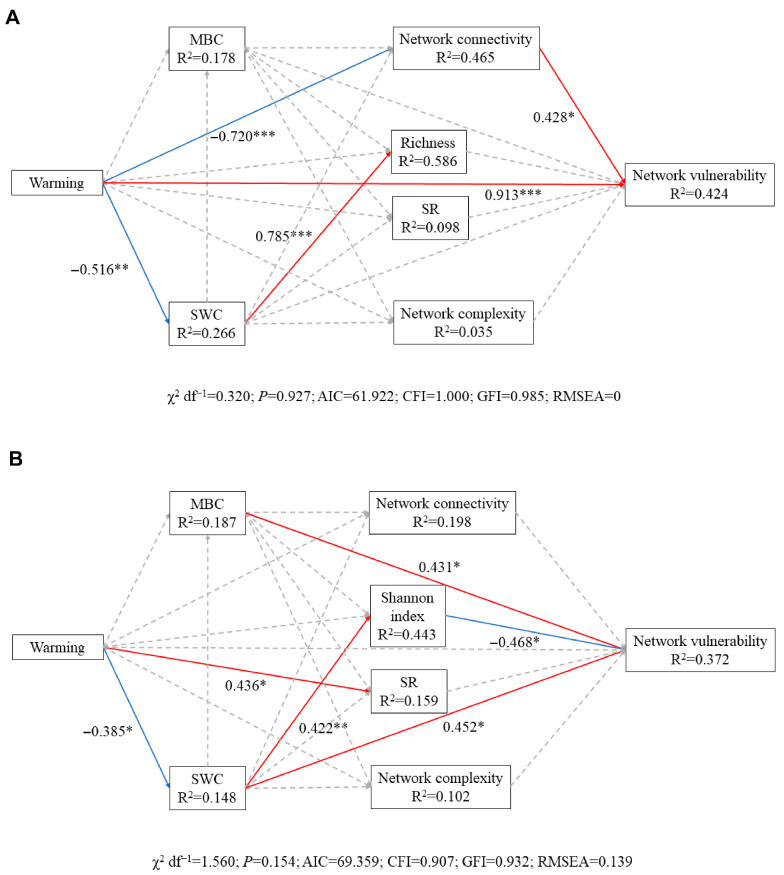
Structural equation modelling (SEM) illustrating the direct and indirect effects of warming on soil moisture, microbial communities, and microbial network properties in (**A**) cyanobacterial and (**B**) moss biocrusts. Positive, negative, and non-significant associations are represented by red, blue, and gray arrows, respectively. Asterisks indicate significance (* *p* < 0.05, ** *p* < 0.01, and *** *p* < 0.001). Numbers adjacent to the pathway arrow denote the standard path coefficients.

The SEMs for both biocrust types showed good model fit (cyanobacterial biocrust: χ^2^ df^−1^ = 0.320; *p* = 0.927, CFI = 1, GFI = 0.985; RMSEA = 0, AIC = 61.922; moss biocrust: χ^2^ df^−1^ = 1.560; *p* = 0.154, CFI = 0.907, GFI = 0.932; RMSEA = 0.139, AIC = 69.359). In cyanobacterial biocrusts (Figure 5A; R^2^ = 0.424), warming had a significant direct positive effect on network vulnerability (β = 0.913). In addition, warming indirectly increased network vulnerability by reducing network connectivity. Moreover, although warming significantly altered soil moisture, it did not appear to, in turn, indirectly affect network vulnerability and complexity. In moss biocrusts (Figure 5B; R^2^ = 0.372), neither network complexity nor vulnerability was influenced by warming directly. Warming affected network vulnerability by altering soil moisture and the Shannon index.

## 4. Discussion

### 4.1. Changes in Soil Microbial Network Complexity and Connectivity in Cyanobacterial and Moss Biocrusts Under Warming

The composition of the soil microbial network showed a higher complexity in cyanobacterial biocrusts compared to moss biocrust (Table 1). This difference was mainly attributable to the lower levels of soil nutrients in cyanobacterial biocrusts (Table S2). In previous research, in low-nutrient environments, microorganisms must intricately regulate their metabolism and resource acquisition strategies to cope with nutrient limitations [65]. Consequently, the composition and activity of microbial communities become more sensitive to environmental variables such as soil moisture and the concentrations of nitrogen, phosphorus, and organic carbon [66]. In such settings, microorganisms rely heavily on efficient resource use and engage in stronger competition [25,31,67], which aligns with the more intricate co-occurrence networks observed here in cyanobacterial biocrusts. In contrast, in the higher-nutrient environment of moss biocrusts, the relative ease of nutrient access appears to reduce the direct influence of these environmental factors on microbial community composition. This does not mean that microbe–environment relationships are absent; rather, microorganisms in nutrient-rich conditions may exhibit a higher adaptive capacity to resource availability, leading to a more buffered response to environmental fluctuations.

Unlike the previous assumption, simulated warming did not significantly affect the complexity of microbial networks in either biocrust type (Table 1). Soil microbial network structure is known to be influenced by both soil physicochemical properties [68] and broader global change factors [69]. Evaluating microbial resistance to warming is critical for predicting how ecosystem function may shift under future climate scenarios [3,70]. Warming-driven changes in soil and plant traits can alter microbial community composition and diversity [71,72,73]. In plant-dominated systems, species-specific root exudates and litter traits mediate the effects of warming on soil microbes [74,75]. In contrast, in biocrust systems with minimal changes to soil properties, these buffering mechanisms may be absent, which may explain the lack of significant change in microbial community complexity despite altered network stability [15].

Network connectivity reflects the strength and density of interactions among nodes within a microbial network [76]. Generally, higher connectivity is associated with greater network density, complexity, stability, and robustness [3]. Our results show that elevated temperatures significantly altered the composition of the microbial co-occurrence network (Figure 2), favoring specialized microbial taxa over generalists (Figure 3). Specifically, warming simplified initially stable and complex microbial ecosystems, leading to decreased phylogenetic diversity (Figure 2), fewer interactions, and lower overall connectivity (Table 1). This simplification may represent a community-level recalibration in response to thermal stress. Consistent with this, our results indicated that non-warming control networks maintained tighter connectivity (Table 1) and higher microbial phylum diversity (Figure 3A, C) than their warming counterparts. Highly connected OTUs are considered beneficial to soil communities irrespective of their specific ecological roles [77]. Simulated warming destabilized these microbial networks, with disproportionate impacts on temperature-sensitive groups like Proteobacteria [78]. Moreover, warming was associated with a slight increase in network modularity (Table 1), which led to more nodes assuming key topological roles and to enhanced interactions between modules [79].

### 4.2. Soil Microbial Network Stability in Cyanobacterial and Moss Biocrusts in Response to Global Change and Biocrust Type

The analysis of co-occurrence networks of soil microbial communities revealed that climate warming reduces microbial network stability in cyanobacterial biocrusts. Our results support the findings of other studies which show that reduced microbial network stability is associated with lower robustness and higher vulnerability [54,55]. However, contrasting findings have been reported, with some studies observing increased microbial network complexity and stability under warming in tallgrass steppes [3], dryland agroecosystems [80], subtropical primary forests [5], and on the Tibetan Plateau [81]. These discrepancies likely reflect variation in ecosystem vulnerability. The Mu Us Sandland is among the world’s most ecologically fragile regions, where even small climatic shifts can have pronounced impacts on ecosystem structure [82]. In such settings, microbial networks may be more susceptible to collapse following warming [83,84]. Another explanation could lie in differences between microbial communities associated with vascular plants and those associated with biocrusts. In vascular plant systems, warming often reduces soil moisture, which can intensify microbial competition and thereby increase network stability [15]. In contrast, in cyanobacterial biocrusts, warming directly reduces network connectivity (Figure 5A), indicating weakened interspecies interactions and, indirectly, less stable networks [3,85].

Our results suggest that moss biocrusts exhibit lower robustness but higher vulnerability than cyanobacterial biocrusts. On the one hand, cyanobacterial biocrusts exhibited greater microbial network stability than moss biocrusts, potentially due to lower moisture and nutrient levels (Table S2), which may intensify microbial competition and stabilize the network [15]. On the other hand, moss biocrusts showed greater resilience to warming, possibly due to their higher microbial OTU richness and Shannon diversity (Figure 2), which could buffer against temperature-induced stress [76]. Moss biocrust microbial communities may also respond more slowly to environmental change, as they have longer turnover times than cyanobacterial biocrusts [86].

Changes in network stability have significant implications for microbial community function [15], highlighting the critical role of microbial interactions in maintaining ecosystem processes [87]. Microbes function within complex ecological networks, where cooperative or interdependent relationships often underpin shared functional roles [88,89]. Thus, our data suggested destabilization of these networks could disrupt cooperative and competitive dynamics, leading to altered functional potential.

### 4.3. Warming Shapes Soil Microbial Keystone Taxa in Cyanobacterial and Moss Biocrusts

Peripheral nodes dominated all four microbial networks (Figure 4), representing specialized taxa with limited external connectivity. This network topology suggests a microbial community adapted to specific local conditions but potentially vulnerable to environmental change, such as warming [90]. We also found that the importance of generalists for maintaining network integrity prior to warming is evident from their higher abundance in control conditions (18 generalists in cyanobacterial biocrust and 12 generalists in moss biocrust) compared to warmed conditions (10 generalists in cyanobacterial biocrust and 4 generalists in moss biocrust) (Figure 4). Generalist and specialist microbes are known to influence microbial community dynamics in distinct ways [91]. A greater abundance of generalists in a network typically promotes stability by facilitating the exchange of energy, information, and materials among species [92]. Generalists possess broad environmental tolerances, whereas specialists are adapted to a narrower range of habitat conditions [93]. Consequently, the marked reduction in generalists following warming is likely detrimental to the microbial network. This decline, coupled with reduced overall microbial diversity and connectivity, could restrict nutrient availability and disrupt key mutualistic interactions that underpin microorganism development. Such changes may ultimately diminish the competitive fitness of microorganisms and increase their susceptibility to environmental stressors.

Warming also led to significant reductions in keystone phyla—those with high connectivity—in biocrust communities (Figure 3). Under ambient conditions, Proteobacteria were the dominant keystone phylum in both biocrust types, playing essential roles in soil stabilization by secreting extracellular polysaccharides that bind sand grains, thereby preventing wind erosion [94]. They are also key nitrogen fixers during early biocrust development [95]. Proteobacteria exhibit strong ecological adaptability and metabolic diversity under resource competition and environmental stress, enabling them to establish close ecological interactions with other microbial groups in extreme environments such as alpine meadows [96]. Under warming, Bacteroidetes became keystone taxa in cyanobacterial biocrusts, contributing to soil aggregation and stability via polysaccharide secretion [97,98,99]. In warmed moss biocrusts, Acidobacteria emerged as keystone taxa, notable for their strong acid and drought tolerance, which enables their survival in harsh surface microenvironments [100,101]. Their presence likely enhances community-level stress tolerance, improving moss biocrust stability under arid acidic conditions. However, declines in Chloroflexi and Cyanobacteria under warming suggest reduced elemental cycling and ecological function [102]. This loss may weaken plant stress resistance [103] and hinder organic matter decomposition [104], further compromising ecosystem resilience.

Microbial hub taxa within co-occurrence networks are likely key regulators of community function and may be crucial for the growth and sustained health of newly established biocrusts. Our study confirmed that such hub taxa and indicator species are present in biocrusts and can be identified using the analytical approaches applied here. However, amplicon sequencing alone provides limited insight into the specific functional guilds or regulatory roles of these taxa. Therefore, future research should focus on elucidating the functional roles of the broader microbial community—including desert soil fungi—using metagenomic technologies, and on their influence on biocrust assembly and stability. This knowledge is essential for designing effective simulation experiments.

One limitation of this study is the data processing method for the ecological network—OTUs clustered at 97%. The field of amplicon data analysis is rapidly evolving, and ASV-based methods (e.g., via the DADA2 pipeline) are now considered the gold standard, offering single-nucleotide resolution and greater comparability across studies than traditional 97% OTU clustering. Future research using ASVs could potentially reveal finer-scale network properties and co-occurrence patterns.

## 5. Conclusions

This study investigated the effects of warming on microbial network complexity, stability, and connectivity in cyanobacterial and moss biocrusts from the Mu Us Sandland. Warming reduced microbial network stability in cyanobacterial biocrusts overall, although cyanobacterial biocrust networks were more stable than those of moss biocrusts. Network complexity remained largely unchanged in both biocrust types. Notably, warming-induced declines in connectivity within cyanobacterial biocrusts destabilized networks, suggesting potential disruptions to ecosystem functions under future climate warming scenarios. Furthermore, warming decreased the number of module hubs and keystone phyla in both biocrust types, leading to fewer keystone taxa and reduced direct microbial interactions. This probably resulted in diminished efficiency of resource and information transfer and impaired ecosystem functionality, driving retrogressive succession characterized by degraded biocrust morphology and function. Collectively, these findings demonstrate that warming-induced alterations in microbial network stability and connectivity could substantially influence biocrust development and succession, with important implications for biogeochemical cycling in semi-arid regions.

## Figures and Tables

**Table 1:** Topological characteristics of microbial networks in cyanobacterial and moss biocrusts under non-warming and warming treatments.

Topological Properties	Cyanobacterial Biocrust		Moss Biocrust	
	Non-Warming	Warming	Non-Warming	Warming
Node	728	672	652	627
Link	959	763	645	584
Percentage of positive links (%)	64.96	64.88	57.83	58.05
R^2^ of power-law fit	0.918	0.910	0.926	0.910
Average degree	2.635	2.271	1.979	1.863
Average clustering coefficient	0.492	0.510	0.356	0.386
Average path length	7.988	10.808	9.253	9.705
Connectivity	0.372	0.207	0.118	0.088
Density	0.059	0.052	0.024	0.021
Modularity	0.853	0.917	0.943	0.955
Diameter	11	10	15	15
Betweenness centralization	0.102	0.078	0.137	0.141
Closeness centrality	0.040	0.033	0.030	0.039
Vulnerability	0.010	0.017	0.016	0.018
Robustness-random removal	0.300	0.288	0.246	0.280
Robustness-targeted removal	0.592	0.520	0.475	0.551
Complexity	0.210	0.211	0.206	0.207

## Data Availability

All data supporting this study are publicly available on the SRA database under BioProject PRJNA714500.

## Supplementary Materials

The following supporting information can be downloaded at: https://www.mdpi.com/article/10.3390/microorganisms14030713/s1, Table S1. Number of clean tags obtained from 16S rRNA gene sequencing and the average percentage of final usable tags for analysis. C: Cyanobacterial crusts; M: Moss crusts; W: Warming; NW: Non-warming. Table S2. Effect of warming on physicochemical characterisation in cyanobacterial and moss biocrusts. Values are means ± standard deviation. Different letters within rows indicate significant differences (*p* < 0.05). Figure S1. Diagram of simulated warming experimental process. Figure S2. Flowchart of bioinformatics analysis for 16S rRNA gene sequencing.

## References

[1] Parry M.L., Canziani O.F., Palutikof J.P., Van Der Linden P.J., Hanson C.E., IPCC (2007). Climate Change 2007: Impacts, Adaptation and Vulnerability. Contribution of Working Group II to the Fourth Assessment Report of the Intergovernmental Panel on Climate Change.

[2] Wang J., Yu C., Fu G. (2021). Warming reconstructs the elevation distributions of aboveground net primary production, plant species and phylogenetic diversity in alpine grasslands. Ecol. Indic..

[3] Yuan M.M., Guo X., Wu L., Zhang Y., Xiao N., Ning D., Shi Z., Zhou X., Wu L., Yang Y. (2021). Climate warming enhances microbial network complexity and stability. Nat. Clim. Change.

[4] Rustad L.E., Campbell J.L., Marion G.M., Norby R.J., Mitchell M.J., Hartley A.E., Cornelissen J.H.C., Gurevitch J., Gcte N. (2001). A meta-analysis of the response of soil respiration, net nitrogen mineralization, and aboveground plant growth to experimental ecosystem warming. Oecologia.

[5] Liu X.D., Ma Q., Yu H., Li Y., Li L., Qi M., Wu W., Zhang F., Wang Y., Zhou G. (2021). Climate warming-induced drought constrains vegetation productivity by weakening the temporal stability of the plant community in an arid grassland ecosystem. Agric. For. Meteorol..

[6] Machado De Lima N.M., Muñoz-Rojas M., Vázquez-Campos X., Branco L.H.Z. (2021). Biocrust cyanobacterial composition, diversity, and environmental drivers in two contrasting climatic regions in Brazil. Geoderma.

[7] Zhao J., Yang W., Tian L., Qu G., Wu G.L. (2024). Warming differentially affects above- and belowground ecosystem functioning of the semi-arid alpine grasslands. Sci. Total Environ..

[8] Bardgett R.D., Freeman C., Ostle N.J. (2008). Microbial contributions to climate change through carbon cycle feedbacks. ISME J..

[9] Zhou J., Xue K., Xie J., Deng Y., Wu L., Cheng X., Fei S., Deng S., He Z., Van Nostrand J. (2012). Microbial mediation of carbon-cycle feedbacks to climate warming. Nat. Clim. Change.

[10] Davidson E.A., Janssens I.A. (2006). Temperature sensitivity of soil carbon decomposition and feedbacks to climate change. Nature.

[11] Tiedje J., Bruns M., Casadevall A., Criddle C., Eloe-Fadrosh E., Karl D., Nguyen N., Zhou J. (2022). Microbes and climate change: A research prospectus for the future. MBio.

[12] Delgado-Baquerizo M., Reich P.B., Trivedi C., Eldridge D.J., Abades S., Alfaro F.D., Bastida F., Berhe A.A., Cutler N.A., Gallardo A. (2020). Multiple elements of soil biodiversity drive ecosystem functions across biomes. Nat. Ecol. Evol..

[13] Ma B., Wang Y., Ye S., Liu S., Stirling E., Gilbert J.A., Faust K., Knight R., Jansson J.K., Cardona C. (2020). Earth microbial co-occurrence network reveals interconnection pattern across microbiomes. Microbiome.

[14] Cheng J., Yang Y., Yuan M.M., Gao Q., Wu L., Qin Z., Shi Z.J., Schuur E.A.G., Cole J.R., Tiedje J.M. (2021). Winter warming rapidly increases carbon degradation capacities of fungal communities in tundra soil: Potential consequences on carbon stability. Mol. Ecol..

[15] Li D., Wu C., Wu J. (2024). Soil fungal community has higher network stability than bacterial community in response to warming and nitrogen addition in a subtropical primary forest. Appl. Environ. Microbiol..

[16] Weber B., Büdel B., Belnap J. (2016). Biological Soil Crusts: An Organizing Principle in Drylands.

[17] Belnap J. (2003). The world at your feet: Desert biological soil crusts. Front. Ecol. Environ..

[18] Garcia-Velazquez L., Gallardo A., Ochoa V., Gozalo B., Lazaro R., Maestre F.T. (2022). Biocrusts increase the resistance to warming-induced increases in topsoil P pools. J. Ecol..

[19] Delgado-Baquerizo M., Morillas L., Maestre F.T., Gallardo A. (2013). Biocrusts control the nitrogen dynamics and microbial functional diversity of semi-arid soils in response to nutrient additions. Plant Soil.

[20] Guan P., Zhang X., Cheng Y., Zheng H., Liang W. (2020). Biocrust regulates the effects of water and temperature on soil microbial and nematode communities in a semiarid ecosystem. Land Degrad. Dev..

[21] Miralles I., Lazaro R., Sanchez-Maranon M., Soriano M., Ortega R. (2020). Biocrust cover and successional stages influence soil bacterial composition and diversity in semiarid ecosystems. Sci. Total Environ..

[22] Miralles I., Trasar-Cepeda C., Soria R., Ortega R., Lucas-Borja M.E. (2021). Environmental and ecological factors influencing soil functionality of biologically crusted soils by different lichen species in drylands. Sci. Total Environ..

[23] Garcia-Pichel F., Wojciechowski M.F. (2009). The evolution of a capacity to build supra-cellular ropes enabled filamentous cyanobacteria to colonize highly erodiblesubstrates. PLoS ONE.

[24] Mora-Montes H.M., Bates S., Netea M.G., Castillo L., Brand A., Buurman E.T., Díaz-Jiménez D.F., Jan Kullberg B., Brown A.J., Odds F.C. (2010). A multifunctional mannosyltransferase family in Candida albicans determines cellwall mannan structure and host-fungus interactions. J. Biol. Chem..

[25] Berry D., Widder S. (2014). Deciphering microbial interactions and detecting keystone species with co-occurrence networks. Front. Microbiol..

[26] Herren C.M., Mcmahon K.D. (2018). Keystone taxa predict compositional change in microbial communities. Environ. Microbiol..

[27] Landi P., Minoarivelo H.O., Brännström Å., Hui C., Dieckmann U. (2018). Complexity and stability of ecological networks: A review of the theory. Popul. Ecol..

[28] Delgado-Baquerizo M., Maestre F.T., Eldridge D.J., Bowker M.A., Ochoa V., Gozalo B., Berdugo M., Val J., Singh B.K. (2016). Biocrust-forming mosses mitigate the negative impacts of increasing aridity on ecosystem multifunctionality in drylands. New Phytol..

[29] Ladron de Guevara M., Gozalo B., Raggio J., Lafuente A., Prieto M., Maestre F.T. (2018). Warming reduces the cover, richness and evenness of lichen-dominated biocrusts but promotes moss growth: Insights from an 8 yr experiment. New Phytol..

[30] Ferrenberg S., Reed S.C., Belnap J. (2015). Climate change and physical disturbance cause similar community shifts in biological soil crusts. Proc. Natl. Acad. Sci. USA.

[31] Faust K., Raes J. (2012). Microbial interactions: From networks to models. Nat. Rev. Microbiol..

[32] de Vries F.T., Griffiths R.I., Bailey M., Craig H., Girlanda M., Gweon H.S., Hallin S., Kaisermann A., Keith A.M., Kretzschmar M. (2018). Soil bacterial networks are less stable under drought than fungal networks. Nat. Commun..

[33] Wagg C., Schlaeppi K., Banerjee S., Kuramae E.E., van der Heijden M.G.A. (2019). Fungal-bacterial diversity and microbiome complexity predict ecosystem functioning. Nat. Commun..

[34] Jia X., Zha T.S., Wu B., Zhang Y.Q., Gong J.N., Qin S.G., Chen G.P., Qian D., Kellomäki S., Peltola H. (2014). Biophysical controls on net ecosystem CO_2_ exchange over a semiarid shrubland in northwest China. Biogeosciences.

[35] Lai Z.R., Zhang Y.Q., Liu J.B., Wu B., Qin S.G., Fa K.Y. (2016). Fine-root distribution, production, decomposition, and effect on soil organic carbon of three revegetation shrub species in northwest China. For. Ecol. Manag..

[36] Feng X., Bu C., Hao H., Yang Y., Zhang G. (2015). Research on biological soil crust extraction by spectral analysis in Mu Us Desert, China. J. Nat. Resour..

[37] Shao C.X. (2015). Species Composition and Nitrogen Dynamics of Biological Soil Crusts in Mu Us Desert. Ph.D. Thesis.

[38] Ju M.C., Zhang T.L., Li X.K., Li B.Y., Li Y.P., Liu Q.Y., Wang Q.X., Bu C.F. (2021). Large scale environmental drivers of biocrust distribution and development across a sandy desert in China. Catena.

[39] Li X., Tian C., Bu C., Gao P., Wu S., Fan J., Zhang W., Pang J., Wei Y., Siddique K.H.M. (2025). Soil Multifunctionality Responses to Warming and Nitrogen Addition and the Mediating Bacteria Vary by Biocrust Type. Eur. J. Soil Sci..

[40] Galic N., Grimm V., Forbes V.E. (2017). Impaired ecosystem process despite little effects on populations: Modeling combined effects of warming and toxicants. Glob. Change Biol..

[41] Risbey J.S., Grose M.R., Monselesan D.P., O’Kane T.J., Lewandowsky S. (2017). Transient response of the global mean warming rate and its spatial variation. Weather. Clim. Extrem..

[42] Tian C., Yue X., Zhou H., Lei Y., Ma Y., Cao Y. (2021). Projections of changes in ecosystem productivity under 1.5 °C and 2 °C global warming. Glob. Planet. Change.

[43] Nelson D.W., Sommer L.E., Page A.L. (1982). Total carbon, organic carbon, and organic matter. Methods of Soil Analysis, Part 2. Chemical and Microbiological Properties.

[44] Crouch S.R., Malmstad H. (1967). A mechanistic investigation of molybdenum blue method for determination of phosphate. Anal. Chem..

[45] Witt C., Gaunt J.L., Galicia C.C., Ottow J.C.G., Neue H.U. (2000). A rapid chloroform-fumigation extraction method for measuring soil microbial biomass carbon andnitrogen in flooded rice soils. Biol. Fertil. Soils.

[46] Bolyen E., Rideout J.R., Dillon M.R., Bokulich N., Abnet C.C., Al-Ghalith G.A., Alexander H., Alm E.J., Arumugam M., Asnicar F. (2019). Reproducible, interactive, scalable and extensible microbiome data science using QIIME 2. Nat. Biotechnol..

[47] Zhou Z., Wang C., Luo Y. (2020). Meta-analysis of the impacts of global change factors on soil microbial diversity and functionality. Nat. Commun..

[48] Cole J.R., Wang Q., Cardenas E., Fish J., Chai B., Farris R.J., Kulam-Syed-Mohideen A.S., McGarrell D.M., Marsh T., Garrity G.M. (2009). The Ribosomal Database Project: Improved alignments and new tools for rRNA analysis. Nucleic Acids Res..

[49] Pruesse E., Quast C., Knittel K., Fuchs B.M., Ludwig W., Peplies J., Gloeckner F.O. (2007). SILVA: A comprehensive online resource for quality checked and aligned ribosomal RNA sequence data compatible with ARB. Nucleic Acids Res..

[50] Deng Y., Jiang Y., Yang Y., He Z., Luo F., Zhou J. (2012). Molecular ecological network analyses. BMC Bioinform..

[51] Sun P.G. (2015). Controllability and modularity of complex networks. Inf. Sci..

[52] Guimerà R., Amaral L.A.N. (2005). Functional cartography of complex metabolic networks. Nature.

[53] Osland M.J., Enwright N.M., Day R.H., Gabler C.A., Stagg C.L., Grace J.B. (2016). Beyond just sea-level rise: Considering macroclimatic drivers within coastal wetland vulnerability assessments to climate change. Glob. Change Biol..

[54] Zhang C., Lei S., Wu H., Liao L., Wang X., Zhang L., Liu G., Wang G., Fang L., Song Z. (2024). Simplified microbial network reduced microbial structure stability and soil functionality in alpine grassland along a natural aridity gradient. Soil Biol. Biochem..

[55] Cornell C.R., Zhang Y., Ning D., Xiao N., Wagle P., Xiao X., Zhou J. (2023). Land use conversion increases network complexity and stability of soil microbial communities in a temperate grassland. ISME J..

[56] Nakayama M., Imamura S., Taniguchi T., Tateno R. (2019). Does conversion from natural forest to plantation affect fungal and bacterial biodiversity, community structure, and co-occurrence networks in the organic horizon and mineral soil?. For. Ecol. Manag..

[57] Byrnes J.E.K., Gamfeldt L., Isbell F., Lefcheck J.S., Griffin J.N., Hector A., Cardinale B.J., Hooper D.U., Dee L.E., Duffy J.E. (2014). Investigating the relationship between biodiversity and ecosystem multifunctionality: Challenges and solutions. Methods Ecol. Evol..

[58] Gamfeldt L., Roger F. (2017). Revisiting the biodiversity-ecosystem multifunctionality relationship. Nat. Ecol. Evol..

[59] Maestre F.T., Quero J.L., Gotelli N.J., Escudero A., Ochoa V., Delgado-Baquerizo M., Garcia-Gomez M., Bowker M.A., Soliveres S., Escolar C. (2012). Plant species richness and ecosystem multifunctionality in global drylands. Science.

[60] Yang K., Zhao Y., Gao L., Sun H., Gu K. (2022). Nonlinear response of hydrodynamic and soil erosive behaviors to biocrust coverage in drylands. Geoderma.

[61] Lefcheck J.S. (2016). PIECEWISESEM: Piecewise structural equation modelling in R for ecology, evolution, and systematics. Methods Ecol. Evol..

[62] Hair J.F.J., Black W.C., Babin B.J., Anderson R.E., Tatham R.L. (2006). Multivariate Data Analysis.

[63] Jones E.B., Hillberry L.E., Jones M.T., Fasihi M., Roushan P., Jiang Z., Ho A.L., Neill C., Ostby E., Graf P. (2022). Small-world complexnetwork generation on a digital quantum processor. Nat. Commun..

[64] Meena C., Hens C., Acharyya S., Haber S., Boccaletti S., Barzel B. (2023). Emergentstability in complex network dynamics. Nat. Phys..

[65] Dai T., Wen D., Bates C.T., Wu L., Guo X., Liu S., Su Y., Lei J., Zhou J., Yang Y. (2022). Nutrient supply controls the linkage between species abundance and ecological interactions in marine bacterial communities. Nat. Commun..

[66] Philippot L., Chenu C., Kappler A., Rillig M.C., Fierer N. (2024). The interplay between microbial communities and soil properties. Nat. Rev. Microbiol..

[67] Lima-Mendez G., Faust K., Henry N., Decelle J., Iudicone D. (2015). Ocean plankton. Determinants of community structure in the global plankton interactome. Science.

[68] Mandakovic D., Rojas C., Maldonado J., Latorre M., Travisany D., Delage E., Bihouee A., Jean G., Diaz F.P., Fernandez-Gomez B. (2018). Structure and co-occurrence patterns in microbial communities under acute environmental stress reveal ecological factors fostering resilience. Sci. Rep..

[69] Ma B., Wang H., Dsouza M., Lou J., He Y., Dai Z., Brookes P.C., Xu J., Gilbert J.A. (2016). Geographic patterns of co-occurrence network topological features for soil microbiota at continental scale in eastern China. ISME J..

[70] Chen X., Tian J., Liu S., Wei Z., Wang Y., Song X., Zhang X., Bai Y. (2022). The complexity of the bacterial community in response to fertilization determines forage production in a semiarid grassland. Ecol. Indic..

[71] Solly E.F., Lindahl B.D., Dawes M.A., Peter M., Souza R.C., Rixen C., Hagedorn F. (2017). Experimental soil warming shifts the fungal community composition at the alpine treeline. New Phytol..

[72] de Oliveira T.B., de Lucas R.C., de Almeida Scarcella A.S., Contato A.G., Pasin T.M., Martinez C.A., Teixeira de Moraes Polizeli M.d.L. (2020). Fungal communities differentially respond to warming and drought in tropical grassland soil. Mol. Ecol..

[73] Nottingham A.T., Scott J.J., Saltonstall K., Broders K., Montero-Sanchez M., Puspok J., Baath E., Meir P. (2022). Microbial diversity declines in warmed tropical soil and respiration rise exceed predictions as communities adapt. Nat. Microbiol..

[74] De L., Jonathan R., Dorrepaal E., Kardol P., Nilsson M.C., Teuber L.M., Wardle D.A. (2016). Contrasting responses of soil microbial and Nematode communities to warming and plant functional group removal across a post-fire boreal forest successional gradient. Ecosystems.

[75] Ma Z., Zhao W., Zhao C., Wang D., Liu M., Li D., Liu Q. (2018). Plants regulate the effects of experimental warming on the soil microbial community in an alpine scrub ecosystem. PLoS ONE.

[76] Zhou H., Gao Y., Jia X., Wang M., Ding J., Cheng L., Bao F., Wu B. (2020). Network analysis reveals the strengthening of microbial interaction in biological soil crust development in the Mu Us Sandy Land, northwestern China. Soil Biol. Biochem..

[77] Chen S., Qi G., Luo T., Zhang H., Jiang Q., Wang R., Zhao X. (2018). Continuous-cropping tobacco caused variance of chemical properties and structure of bacterial network in soils. Land Degrad. Dev..

[78] Hernandez D.J., David A.S., Menges E.S., Searcy C.A., Afkhami M.E. (2021). Environmental stress destabilizes microbial networks. ISME J..

[79] He D., Shen W., Eberwein J., Zhao Q., Ren L., Wu Q.L. (2017). Diversity and co-occurrence network of soil fungi are more responsive than those of bacteria to shifts in precipitation seasonality in a subtropical forest. Soil Biol. Biochem..

[80] Qi J.J., Gao M., Peng Z.H., Pan H.B., Chen S., Lu M.M. (2025). Microbial community resistance is associated with soil carbon degradation under warming condition in dryland agroecosystems. Appl. Soil Ecol..

[81] Chen W., Zhou H., Wu Y., Li Y., Qiao L., Wang J., Zhai J., Song Y., Zhao Z., Zhang Z. (2021). Plant-mediated effects of long-term warming on soil microorganisms on the Qinghai-Tibet Plateau. Catena.

[82] Feng X., Fu B., Piao S., Wang S., Ciais P., Zeng Z., Lu Y., Zeng Y., Li Y., Jiang X. (2016). Revegetation in China’s Loess Plateau is approaching sustainable water resource limits. Nat. Clim. Change.

[83] Ullah H., Nagelkerken I., Goldenberg S.U., Fordham D.A. (2018). Climate change could drive marine food web collapse through altered trophic flows and cyanobacterial proliferation. PLoS Biol..

[84] Zhang J., Zhang B., Liu Y., Guo Y., Shi P., Wei G. (2018). Distinct large-scale biogeographic patterns of fungal communities in bulk soil and soybean rhizosphere in China. Sci. Total Environ..

[85] Wu M.H., Chen S.Y., Chen J.W., Xue K., Chen S.L., Wang X.M., Chen T., Kang S.C., Rui J.P., Thies J.E. (2021). Reduced microbial stability in the active layer is associated with carbon loss under alpine permafrost degradation. Proc. Natl. Acad. Sci. USA.

[86] Tian C., Pang J., Bu C., Wu S., Bai H., Li Y., Guo Q., Siddique K.H.M. (2023). The microbiomes in lichen and moss biocrust contribute differently to carbon and nitrogen cycles in arid ecosystems. Microb. Ecol..

[87] Xue P., Minasny B., McBratney A.B. (2022). Land-use affects soil microbial co-occurrence networks and their putative functions. Appl. Soil Ecol..

[88] Cremer J., Melbinger A., Wienand K., Henriquez T., Jung H., Frey E. (2019). Cooperation in microbial populations: Theory and experimental model systems. J. Mol. Biol..

[89] Deshpande R., VanderSluis B., Myers C.L. (2013). Comparison of profile similarity measures for genetic interaction networks. PLoS ONE.

[90] Yu H., Ren G., Huang Z., Qi S., Zhao B., Fan X., Zhu Z., Dai Z., Du D. (2024). Effects of Increasing Temperature on Bacterial Community Diversity in Mixed Stands of *Artemisia argyi* and *Solidago canadensis* in Eastern China. Microorganisms.

[91] Pandit S.N., Kolasa J., Cottenie K. (2009). Contrasts between Habitat Generalists and Specialists: An Empirical Extension to the Basic Metacommunity Framework. Ecology.

[92] Olesen J.M., Bascompte J., Dupont Y.L., Jordano P. (2007). The Modularity of Pollination Networks. Proc. Natl. Acad. Sci. USA.

[93] Xu Q., Vandenkoornhuyse P., Li L., Guo J., Zhu C., Guo S., Ling N., Shen Q. (2022). Microbial Generalists and Specialists Differently Contribute to the Community Diversity in Farmland Soils. J. Adv. Res..

[94] Gundlapally S.R., Garcia-Pichel F. (2006). The community and phylogenetic diversity ofbiological soil crusts in the Colorado plateau studied by molecular fingerprinting andintensive cultivation. Microb. Ecol..

[95] Pepe-Ranney C., Koechli C., Potrafka R., Andam C., Eggleston E., Garcia-Pichel F., Buckley D.H. (2016). Non-cyanobacterial diazotrophs mediate dinitrogen fixation inbiological soil crusts during early crust formation. ISME J..

[96] Baldrian P., Head I.M., Prosser J.I., Schloter M., Smalla K., Tebbe C.C. (2011). Ecology and metagenomics of soil microorganisms. FEMS Microbiol. Ecol..

[97] García-Palacios P., Vandegehuchte M.L., Shaw E.A., Dam M., Post K.H., Ramirez K.S., Sylvain Z.A., de Tomasel C.M., Wall D.H. (2015). Are there links between responses of soil microbes and ecosystem functioning to elevated CO_2_, N deposition and warming? A global perspective. Glob. Change Biol..

[98] Bradford M.A., Davies C.A., Frey S.D., Maddox T.R., Melillo J.M., Mohan J.E., Reynolds J.F., Treseder K.K., Wallenstein M.D. (2008). Thermal adaptation of soil microbial respiration to elevated temperature. Ecol. Lett..

[99] Kanzaki Y., Takemoto K. (2021). Diversity of dominant soil bacteria increases with warming velocity at the global scale. Diversity.

[100] Tian C., Xi J., Ju M., Li Y., Guo Q., Yao L., Wang C., Lin Y., Li Q., Williams W.J. (2021). Biocrust microbiomes influence ecosystem structure and function in the Mu Us Sandland, northwest China. Ecol. Inform..

[101] Pankratov T.A., Ivanova A.O., Dedysh S.N., Liesack W. (2011). Bacterial populations and environmental factors controlling cellulose degradation in an acidic Sphagnum peat. Environ. Microbiol..

[102] Liu J., Cui X., Liu Z., Guo Z., Yu Z., Yao Q., Sui Y., Jin J., Liu X., Wang G. (2019). The Diversity and Geographic Distribution of Cultivable Bacillus-Like Bacteria Across Black Soils of Northeast China. Front. Microbiol..

[103] Sazykin I., Khmelevtsova L., Azhogina T., Sazykina M. (2023). Heavy metals influence on the bacterial community of soils: A review. Agriculture.

[104] Fu J., Xiao Y., Wang Y.F., Liu Z.H., Yang K.J. (2019). Trichoderma affects the physiochemical characteristics and bacterial community composition of saline-alkaline maize rhizosphere soils in the cold-region of Heilongjiang Province. Plant Soil.

