# Supplementary material for: The Microbial Network Stability in Cyanobacterial and Moss Biocrusts Respond Differently to Climate Warming
Source: Microorganisms. 2026 Mar 22;14(3):713. doi: 10.3390/microorganisms14030713 (PMC13029624; doi:10.3390/microorganisms14030713)
Supplement: Supplementary file 1 [file microorganisms-14-00713-s001.zip › microorganisms-4126238-supplementary.pdf]

Table S1. Number of clean tags obtained from 16S rRNA gene sequencing and the average percentage of final usable tags for analysis. C: Cyanobacterial crusts; M: Moss crusts; W: Warming; NW: Non-warming.

Sample	Clean_tags	Final_tags	Percentage (%)	Sample	Clean_tags	Final_tags	Percentage (%)
NW-C-1	90457	38847	42.9	NW-M-1	77242	38847	50.3
NW-C-2	90148	38847	43.1	NW-M-2	87082	38847	44.6
NW-C-3	86572	38847	44.9	NW-M-3	89820	38847	43.2
NW-C-4	89134	38847	43.6	NW-M-4	90065	38847	43.1
NW-C-5	89969	38847	43.2	NW-M-5	106353	38847	36.5
NW-C-6	86999	38847	44.7	NW-M-6	83173	38847	46.7
NW-C-7	86941	38847	44.7	NW-M-7	80215	38847	48.4
NW-C-8	87152	38847	44.6	NW-M-8	88183	38847	44.1
NW-C-9	89450	38847	43.4	NW-M-9	85999	38847	45.2
NW-C-10	87640	38847	44.3	NW-M-10	90468	38847	42.9
NW-C-11	87985	38847	44.2	NW-M-11	88990	38847	43.7
NW-C-12	86231	38847	45.0	NW-M-12	84495	38847	46.0
NW-C-13	86980	38847	44.7	NW-M-13	90333	38847	43.0
NW-C-14	90080	38847	43.1	NW-M-14	90476	38847	42.9
NW-C-15	89615	38847	43.3	NW-M-15	86315	38847	45.0
W-C-1	85597	38847	45.4	W-M-1	87300	38847	44.5
W-C-2	88851	38847	43.7	W-M-2	85775	38847	45.3
W-C-3	86666	38847	44.8	W-M-3	88524	38847	43.9
W-C-4	88175	38847	44.1	W-M-4	87807	38847	44.2
W-C-5	84452	38847	46.0	W-M-5	91242	38847	42.6
W-C-6	85506	38847	45.4	W-M-6	88365	38847	44.0
W-C-7	90349	38847	43.0	W-M-7	88713	38847	43.8
W-C-8	91775	38847	42.3	W-M-8	86095	38847	45.1
W-C-9	87994	38847	44.1	W-M-9	77294	38847	50.3
W-C-10	87161	38847	44.6	W-M-10	81493	38847	47.7
W-C-11	88191	38847	44.0	W-M-11	84617	38847	45.9
W-C-12	84944	38847	45.7	W-M-12	85568	38847	45.4
W-C-13	85777	38847	45.3	W-M-13	86438	38847	44.9
W-C-14	87387	38847	44.5	W-M-14	71833	38847	54.1
W-C-15	86607	38847	44.9	W-M-15	71809	38847	54.1

Table S2. Effect of warming on physicochemical characterisation in cyanobacterial and moss biocrusts. Values are means ± standard deviation. Different letters within rows indicate significant differences (p < 0.05).

Treatment	Cyanobacterial biocrust		Moss biocrust	
	Non-warming	Warming	Non-warming	Warming
Soil moisture (%)	1.15±0.62a	0.63±0.11b	19.57±3.00a	16.62±4.21b
pH	7.29±0.05b	7.37±0.09a	7.33±0.09a	7.29±0.11a
SOC ($g \cdot kg^{-1}$)	5.82±1.11a	4.87±1.55a	14.87±2.28b	18.59±1.11a
TN ($g \cdot kg^{-1}$)	0.43±0.09a	0.35±0.11b	1.20±0.27b	1.53±0.11a
TP ($g \cdot kg^{-1}$)	0.25±0.03a	0.25±0.02a	0.38±0.04b	0.43±0.02a
MBC ($mg \cdot kg^{-1}$)	167.06±50.75a	129.67±40.37b	455.90±109.13a	519.75±79.04a
MBN ($mg \cdot kg^{-1}$)	15.50±4.29a	7.66±1.44b	43.94±16.05a	49.24±7.55a

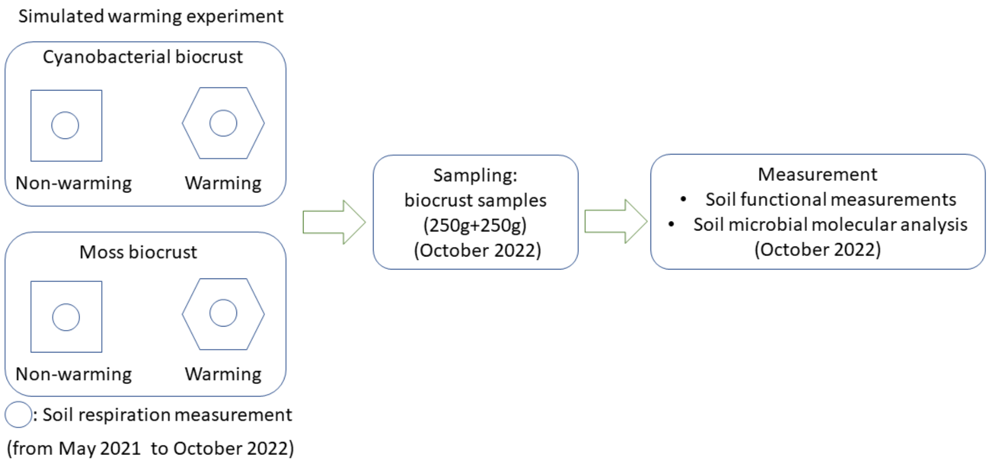

Figure S1. Diagram of simulated warming experimental process.

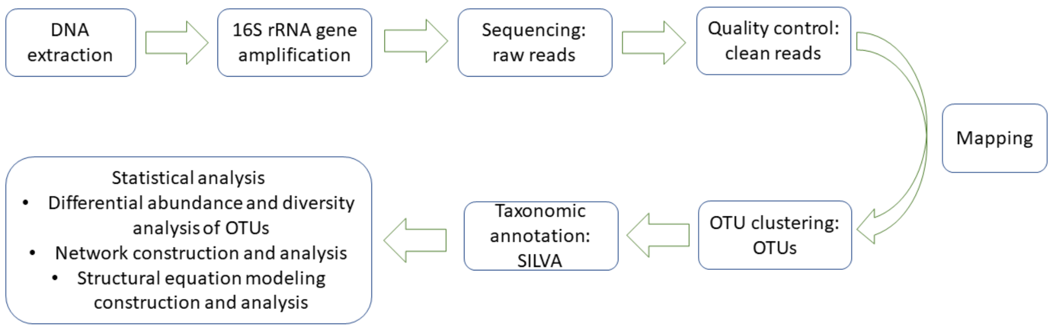

Figure S2. Flowchart of bioinformatics analysis for 16S rRNA gene sequencing.
